# Treating seizures and preventing amnesia in LGI1-antibody encephalitis: A new MRI signature?

**DOI:** 10.1212/NXI.0000000000000182

**Published:** 2015-11-24

**Authors:** James A. Varley, Sarosh R. Irani

**Affiliations:** From the Nuffield Department of Clinical Neurosciences, University of Oxford, Oxford, UK.

Leucine-rich glioma-inactivated 1 (LGI1)-antibody encephalitis is a treatable disease within the ever-expanding group of autoimmune encephalitides. The illness is typically characterized by the subacute onset of amnesia, confusion, and seizures in middle age, with approximately 60% of patients showing medial temporal lobe T2 hyperintensities.^1,2,e1,e2^ The most characteristic seizure syndrome associated with the LGI1 antibody is the recently termed entity of faciobrachial dystonic seizures (FBDS).^3–5^ FBDS consist of brief, frequent dystonic movements that most commonly affect the arm and ipsilateral hemiface. They occur an average of 50 times per day and can affect the leg and trunk. The leg involvement appears to be a hitherto overlooked cause of falls.^e3^ Of importance, FBDS appear to respond preferentially to immunotherapies over antiepileptic drugs.

In this issue of *Neurology® Neuroimmunology & Neuroinflammation*, Flanagan et al.^6^ focus on a novel imaging finding that they observed in patients with FBDS and LGI1 antibodies but not in patients with LGI1-antibody encephalitis without FBDS. They began by searching a database over a 13-year period, and they identified 89 patients with raised levels of voltage-gated potassium channel complex (VGKC-complex) antibodies. Forty-eight of these had antibodies to LGI1 determined by a fixed cell-based assay (CBA). Twenty-six of the 48 were clinically defined as having FBDS and are the focus of their study.

Indeed, clinical recognition of FBDS is paramount. On detailed questioning, we have found that patients with FBDS may show loss of awareness and speech arrest associated with episodes, as well as sensory auras, postictal confusion, and manual automatisms—all features consistent with seizures. The diagnosis may be overlooked because >90% of ictal EEGs are normal during seizures, CSF is often noninflammatory, and consistent MRI abnormalities are not recognized. Although the syndrome of FBDS is becoming more ingrained in clinical neurology, a striking observation by Flanagan et al was the high proportion (38%) initially diagnosed with psychiatric or functional disorders and the 19% who were suspected of having Creutzfeldt-Jakob disease.^7^

In addition to diagnosis, the treatment and timing of FBDS are of clinical importance. Previous retrospective and prospective observations have shown that immunotherapies produce a more marked reduction in FBDS than antiepileptic drugs. Furthermore, there appears to be an emerging temporal trend, with onset of FBDS followed by the development of cognitive impairment (CI) in about 60% of cases.^3,4,e4^ The corresponding figure was 67% in the study by Flanagan et al. Furthermore, a small prospective study suggested that it may be possible to prevent subsequent CI with effective treatment of FBDS.^3^

In the context of these emerging therapeutic implications, the article by Flanagan et al. describes a potentially important imaging correlate to assist in the diagnosis of FBDS. The authors show a novel pattern of basal ganglia (BG) T1 and/or T2 hyperintensities in 11 of 26 patients with LGI1 antibodies and FBDS. Ten patients were described as displaying unilateral T1 hyperintensities, generally contralateral to FBDS, at various time points. Eight of the 10 had accompanying T2 hyperintensities. One other patient had isolated BG T2 hyperintensities. On average, T1 hyperintensities lasted 11 weeks vs 1 week for T2 hyperintensities. Five lesions showed restricted diffusion on diffusion-weighted imaging, and 2 patients went on to develop caudate atrophy. None of the LGI1 antibody–positive patients without FBDS showed these BG imaging abnormalities.

Previous cohort studies reported less frequent BG T2 hyperintensities,^3,4^ and perhaps dedicated reading of images by neuroradiologists improved the rate of detection seen by Flanagan et al. Alternatively, serial imaging timings within individual patients or the sequences acquired may account for this difference. Nevertheless, BG abnormalities have been reported using a variety of imaging modalities in FBDS patients, and include changes in PET, SPECT and contrast uptake images.^3,4,8–10,e5,e6^

As the authors acknowledge, this is a retrospective study with nonstandardized timing of scans and variable and often short durations of follow-up. This means we cannot draw firm conclusions about precisely when the abnormalities appear or disappear. We can, however, be more confident that the T1 changes persist significantly longer than the T2 abnormalities. Also, compared to a live CBA, the fixed LGI1-antibody CBA used in this study can fail to detect some patients with low levels of LGI1 antibodies (S.R.I., unpublished data); of interest, Flanagan et al. noted 4 patients with FBDS and VGKC-complex antibodies but without LGI1 specificity.

The T1 hyperintensities are particularly intriguing, not least their pathophysiology. The authors suggest a comprehensive list of causes for T1 hyperintensities. They suggest that hypoxic damage is the most likely substrate, providing a novel mechanism for neurotoxicity in autoimmune encephalitis. This could result from either immune-mediated BG damage or high seizure frequency leading to neuronal metabolic stress, excitotoxicity, and consequent neuroglial cell disruptions within the neural network responsible for FBDS.

This neural network is likely to engage the BG. The prominent dystonic posturing seen in FBDS, along with frequently normal ictal EEGs, have led to the suspicion that these episodes are subcortical in origin. In addition, the accumulating imaging findings described above further corroborate this hypothesis. However, the BG could be a consistent part of the network through which seizure activity propagates, rather than the primary epileptogenic focus. If the dystonic episodes seen in FBDS are indeed best described as BG seizures, it raises the larger question of how many other paroxysmal movement disorders are mediated by subcortical epileptic activity.^e7^

In summary, recognition of this novel and unusual imaging appearance may allow for diagnosis and prompt treatment of FBDS, with amelioration of seizures and possible prevention of CI. Neuroradiologists should be asked specifically about these findings in the correct clinical context. Future studies should focus on determining the temporal relationship between these imaging changes and clinical observations and exploring their underlying mechanisms.
